# Self-Cross-Linked Hyaluronic Acid Gel for Adhesion Prophylaxis in Laparoscopic Deep Endometriosis Removal: Safety Report of a Prospective Pilot Study

**DOI:** 10.3390/jcm13206284

**Published:** 2024-10-21

**Authors:** Maya Sophie de Wilde, Rajesh Devassy, Harald Krentel, Rudy Leon De Wilde, Luz Angela Torres-de la Roche

**Affiliations:** 1 University Hospital for Gynecology Pius Hospital, University Medicine Oldenburg, 26121 Oldenburg, Germanyrudy-leon.dewilde@pius-hospital.de (R.L.D.W.); 2 Centre of Excellence in Gynecological Minimal Access Surgery and Oncology, Dubai London Clinic & Specialty Hospital, Dubai 337150, United Arab Emirates; 3 Clinic of Gynecology, Obstetrics, Oncology and Senology, Bethesda Hospital, 47053 Duisburg, Germany; krentel@cegpa.org

**Keywords:** hyaluronic acid gel, barrier, adhesion prophylaxis, deep endometriosis, laparoscopic surgery

## Abstract

**Background/Objectives:** Surgical removal of deep endometriosis lesions is an established method of reducing patient symptoms, but it often results in iatrogenic adhesions that lead to further problems. This pilot study presents the safety evaluation of a novel self-cross-linked hyaluronic acid gel used to reduce adhesions after non-bowel deep endometriosis surgery. **Methods:** A single cohort, single-center, non-randomized pilot study was conducted in patients diagnosed with non-bowel deep endometriosis who underwent a three-stage treatment regimen consisting of first surgery, hormone therapy and second surgery. The present report is limited to an analysis of the inflammatory parameters, pain and complications occurring within a 72 h period following the initial laparoscopy (FLL) utilizing the anti-adhesion gel. **Results:** 60 patients (28.48 ± 5.9 years old) were included. 24 h after the intervention, a slight elevation in C-reactive protein levels was observed in 38.33% of cases (0.98 ± 1.46 mg/dL), with a statistically significant difference after FLL (0.98 ± 1.46 mg/dL before FLL vs. 1.03 ± 1.29 mg/dL after FLL; *p* =< 0.001); there were no patients with levels above 10 mg/dL before or after surgery. 24 h after FLL, 29.33% of patients had a leukocyte count greater than 11 Thous/μL, with a maximum observed value of 16.2 Thous/μL. The count was found to be statistically significantly higher after FLL (6.03 ± 1.91 Thous/μL before FLL vs. 9.15 ± 2.61 Thous/μL after FLL; *p* =< 0.001). At 72 h post-intervention, postoperative pain was reported in up to 63.33% of cases, and one urinary tract infection with fever occurred but was not considered to be related to the product. No serious adverse events were observed. **Conclusions:** The results of this exploratory study showed a safe range of inflammatory response within a 24 h period following the application of the novel self-cross-linked hyaluronic acid antiadhesion gel (HyaRegen^®^) in patients who underwent laparoscopic surgery for non-bowel deep endometriosis.

## 1. Introduction

Iatrogenic adhesions are a common complication after surgical procedures that can result in chronic pain, subfertility, and possible intestinal obstruction [1]. Adhesions can also form because of endometriosis inflammatory diseases of trauma, whether surgically or otherwise [1,2,3]. Endometriosis is a disease that affects an estimated 200 million women worldwide, but the specific probability of adhesion formation by the endometriosis disease itself cannot be statistically accurately predicted. While one study stated that 74% of patients had adhesions during the first operation [4], another study calculated a prevalence of 37.6%, with 51.2% adnexal, followed by 24.4% to the abdominal wall [5]. The surgical excision of deep endometriotic lesions is a well-established method for alleviating patient symptoms. However, during abdominopelvic surgery, the probability of developing postoperative adhesions is estimated to be between 20% and 93%; laparoscopic procedures are not excluded [6]. The local inflammatory response induced by endometriotic foci, or by use of surgical material, disrupts the normal molecular and gene expression signatures during the peritoneal repair process, resulting in an imbalance in the fibrinolysis process and a permanent fibrin matrix [7,8]. The utilization of anti-adhesion agents has been proposed as a means of mitigating this risk [9]. Nevertheless, there is a paucity of evidence supporting the safety and efficacy of natural and synthetic hydrogels following non-bowel deep endometriosis surgery.

A number of anti-adhesive barrier agents (powders, gels, liquids or film-forming materials) are available throughout the world, with only a limited effective role in the pathogenesis of the development of an adhesion. New products are being developed by adding molecular modifications, anti-inflammatory or antioxidant substances or biomaterials, seeking to combine both physical barrier and pharmacotherapeutic strategies, with the aim of improving the handling, metabolic degradation, and efficacy of the former agents [10,11,12]. Examples of newly tested antiadhesion substances are quercetin [7] and epigallocatechin-3-gallate, which have been shown to decrease oxidative stress, inflammation, and fibrosis [13]. Carboxymethyl chitosan has been shown to have analgesic and bacteriostatic effects, and to promote hemostasis and wound healing [14]; anti-fibrotic and anti-angiogenic properties have been reported for endostatin [7]. Biomaterials like F127 and cross-linked glyoxal are used to enhance the mechanical properties of the hydrogels [7,15].

On the other hand, few studies have reported the efficacy of new products in terms of fertility and pain relief in endometriosis patients. A recent RCT showed that a potato starch-based product was able to reduce adhesions by 85% in a small cohort of patients with deep infiltrating endometriosis (DIE), as well as cycle-independent pelvic pain and dysmenorrhoea, with a significantly higher pregnancy rate in the intervention group (64% vs. 21%) [16]. The novel adhesion prophylactic agent (HyaRegen^®^ Gel—BioRegen Biomedical Co., Changzhou, China) evaluated in this study, contains self-cross-linked hyaluronic acid, which differs from similar agents because it is a non-animal-sourced hyaluronic acid raw material, with increased viscosity and hydrophilic properties, completely absorbable 7 to 14 days after application [17]. 

In recent small-sized studies, this gel has been reported to be safe and effective in reducing the postoperative incidence of moderate or severe adnexal and abdominal adhesions [17] as well as pain, with improvements in the physical–mental quality-of-life of patients after deep-infiltrating endometriosis (DIE) surgery [18]. To enable other working groups conducting scientific studies in this field, the present report is limited to an analysis of the inflammatory parameters, pain, and complications occurring within a 24 h period following the initial surgical laparoscopy utilizing the novel anti-adhesion gel.

## 2. Materials and Methods

A single-cohort, single-center, non-randomized study was conducted with consecutively screened women aged 18 to 55 years who underwent laparoscopy for the treatment of DIE. This pilot, explorative study entitled “Assessment of the anti-adhesion effectiveness of HyaRegen^®^ in laparoscopic deep endometriosis surgery: Achyles study” was carried out as an obligatory first step towards a future randomized controlled trial (RCT).

The study population consisted of adult women who were suspicious of DIE and who had provided informed consent prior to surgery, which was enrolled in the study intraoperatively, following a confirmation of the diagnosis of non-bowel deep endometriosis; this was in accordance with the revised American Society for Reproductive Medicine (rASRM) score and the Enzian classification [19]. Patients underwent a three-stage therapy consisting of the first-look diagnostic and surgical laparoscopy (FLL) with excision of the deep endometriotic foci and application of the antiadhesion gel, the administration of a GnRH-analog for a period of 12 to 32 weeks, and a subsequently second-look laparoscopy (SLL) [20]. The last was performed to eradicate any residual endometriosis or adhesions [1,9,10]. The effect of the applied hyaluronic acid gel on adhesiogenesis was examined during the SLL and will be published in the near future. The present report is limited to an analysis of the inflammatory parameters and pain complications within a 72 h period following the FLL.

The adhesion prophylactic agent evaluated in this study (CE 0123) was sealed in glass syringes (10 and 20 mL) and had a concentration of 5 mg/mL, as the application should not exceed 2 ml/kg body weight. The intraperitoneal route of administration is made by connecting the syringe to a plastic cannula provided in the sterile packaging and should involve coating the operated organ and the tissue surface where the surgical trauma or adhesiolysis occurred, as well as adjacent and possible adhesiogenic surfaces.

Safety parameters analyzed included C-reactive protein (CRP) levels, and leukocyte counts taken before and 24 h after the application of the gel during FLL. At 72 h post-intervention, pain and adverse effects were assessed using a visual analog scale (range 0 = absent to 10 = max) and clinical presentation, respectively. In the context of local laboratory norms, a CRP value below 0.5 mg/dL and a leukocyte count within the range of 3800 to 10,500 per microliter of blood (Thous/μL) are considered to indicate a state of normality in women. Furthermore, an intraoperative assessment was conducted to evaluate the handling aspects of the application cannula and gel dispersion on the deperitonealized area resulting from the excision of endometriosis foci, as reported by surgeons during the application procedure.

The sample size was calculated by the Institute for Biostatistics at the Westphalian Wilhelms University of Münster (power = 80%; McNemar test). In order to achieve the initial indication and estimation accuracy with regard to safety and adhesion prevention. The number of cases was increased from the initially planned 20 to a total of 70 following the absence of any serious severe adverse event (SAE) after the first 20 surgeries. Categorical variables are presented as numbers and percentages. Continuous variables are presented as means or standard deviations, as appropriate. Comparisons of safety variables before and after FLL were made using the long-range test. A *p*-value of less than 0.05 was determined to indicate statistically significant differences in the safety parameters prior to and following FLL.

This pilot study was conducted at the University Hospital for Gynecology, Pius Hospital Oldenburg, which is a leading research center for the study of adhesion and endometriosis. It is a Clinical Science Endometriosis Center Level 3 (SEF, EEL). Approval was granted by the Medical Ethics Committee of the Carl von Ossietzky University of Oldenburg (reference number 125-2020). Prior to the surgical procedure, all patients had been provided with the relevant information and had signed an informed consent form. The study was conducted in accordance with the ethical guidelines that govern research in this field. The ethics committee required that this pilot study be conducted as a prerequisite for the future implementation of a properly designed randomized controlled trial.

## 3. Results

Of the 70 patients who provided consent, 60 were diagnosed intraoperatively with non-bowel deep endometriosis and subsequently underwent excision of the endometriosis foci, followed by the application of the antiadhesion gel. Pain, adverse events, and inflammatory parameters reported after FLL are presented in Table 1. Postoperative pain was reported in 63.33% (38/60) of patients, with 61.67% (37/60) experiencing abdominal pain. One patient (1/60) experienced a urinary tract infection with fever, which was deemed unlikely to be related to the product. No other adverse effects were reported. Elevated levels of CRP and leukocytes were observed in 38.33% (23/60) and 33.33% (20/60) of patients, respectively.

A statistically significant difference (*p* = < 0.001) was observed in CRP values before and after FLL (Table 2), with slightly elevated mean CRP levels after FLL (0.98 ± 1.46 mg/dL before FLL vs. 1.03 ± 1.29 mg/dL after FLL). After surgery, 14 patients had CRP levels between 1.0 and 10.0 mg/dL, whereas there were no patients with CRP levels above 10 mg/dL before or after FLL.

The leukocyte counts prior to and following FLL are shown in Table 3. Leukocytosis is typically defined as a white blood cell count exceeding 11 Thous/μL. The leukocyte count was found to be statistically significantly higher after FLL (6.03 ± 1.91 Thous/μL before FLL vs. 9.15 ± 2.61 Thous/μL after FLL; (*p* = < 0.001). Following FLL, 14 patients (29.33%) exhibited leukocyte levels exceeding 11 Thous/μL, with a maximum observed value of 16.2 Thous/μL.

Regarding the handling of the gel, it is recommended to connect the pre-filled syringe containing the gel to the applicator cannula and then gently eject the gel to cover the selected site. It is expected that the gel will adhere easily to applied surfaces. Minor difficulties were observed and reported by the surgeons during this step. In 10% (n = 6/60) of cases a sporadic detachment of the applicator from the syringe was noticed during the application, and some surgeons found that the plastic applicator was relatively firm, requiring some force to bring the applicator to the desired spot, resulting in a reduction in fine motoric laparoscopic movement during injection (16%; n = 10/60). In approximately one-third of cases (31.2%, n = 19/70), a gravity-induced downward flow was observed when the application was performed on the ventral peritoneum spots.

## 4. Discussion

The future of research into adhesion prophylaxis is moving towards the development of more effective and manageable substances. These could facilitate a reduction in the burden of long-term complications associated with postsurgical adhesions in gynecological surgery, thereby offering surgeons valuable aid in this field [21,22]. The analysis presented here responds to the necessity to assess the short-term safety profile of a new-cross-linked hyaluronic acid gel (HyaRegen^®^ Gel) when applied to deperitonealized areas left after the excision of DIE lesions. This profile was examined by monitoring pain scale, CRP and leukocyte counts 24 h after surgery. In particular, the postoperative CRP level is an affordable diagnostic parameter for inflammation. It serves to reflect the potential consequences of surgical trauma or substance-related side effects. In most healthy adults, the normal CRP value is below 0.3 mg/dL. A slight increase in values between 0.3 mg/dL and 1.0 mg/dL can be caused by factors such as obesity, pregnancy, depression, diabetes, colds or cigarette smoking. Values above 1.0 mg/dL, on the other hand, can be seen as a sign of systematic inflammation and above 10 mg/dL can be a sign of an acute bacterial or viral infection [23]. It is expected that inflammatory and pro-inflammatory markers remain within safe limits since an exaggerated elevation could reflect a disruption of the wound healing process and, thus, the high possibility of adhesion formation [24]. Interestingly, there are only a few studies on the safety of anti-adhesive agents in which pain and inflammatory reactions are investigated [25,26].

In the context of DIE surgical techniques that induce an exaggerated inflammatory response, the use of energy is a factor that warrants consideration. The extended thermal effect of monopolar and bipolar diathermy, two widely utilized coagulation methods in DIE laparoscopic surgery, has the potential to induce adhesion formation and diminish ovarian reserve following endometrioma surgery [27,28]. In order to mitigate these risks, ultrasound-guided aspiration of the endometrioma, the most frequent form of DIE [29], followed by chemical sclerotherapy of the cyst plaque, has been proposed as a means of reducing the aforementioned risks. A growing body of evidence from multiple studies indicates that this procedure is safe and effective, with no adverse effects on ovarian function [30,31]. Reviews and meta-analyses of the efficacy and safety of transvaginal ethanol sclerotherapy [30,31] revealed that the majority of patients (85%) experienced significant pain relief. While few cases reported complete symptom resolution, a small percentage (mean 12%) of cases reported minor complications, including abdominal pain, pelvic discomfort, abnormal bleeding or fever. Moreover, the levels of pro-inflammatory markers, namely cytokines IL-1β and IL-6, which have been demonstrated to be elevated in endometrioma patients in comparison to healthy subjects, remained unaltered following sclerosis therapy [32]. Nevertheless, the evidence regarding the recurrence rate of endometrioma remains inconclusive, and there is a lack of evidence concerning serum CRP level changes after the procedure [30,31].

The current study found that all patients exhibited a modest elevation in both postoperative inflammatory parameters (C-reactive protein and white blood cell count), which were markedly elevated in comparison to pre-operative levels. However, the C-reactive protein levels remained below 10 mg/dL. That is, the elevation was not associated with peri-operative infections. Similarly, as previously reported by our research group, a significant elevation in CRP values was observed following the administration of a starch-potato-based antiadhesion product (4DryField® PH, PlanTec Medical GmbH, Lüneberg, Germany) in women who underwent laparoscopic adhesiolysis plus gynecological surgery (87 vs. 29%; *p* < 0.001), when compared to the non-intervention group; the leukocyte levels of all patients remained within the normal range [33].

The occurrence of other forms of local inflammatory reactions following the administration of anti-adhesive agents to prevent mechanical contact between serosal surfaces during the healing peritoneal period following abdominopelvic surgery has been documented in a limited number of studies. The presence of foreign body granulomas was confirmed histopathologically at the sites where residues of some barriers were observed during the second-look surgery [34,35,36], particularly following the application of the oxidized regenerated cellulose barrier (Gynecare Interceed®, Ethicon-Johnson & Johnson, Neuss, Germany), the hyaluronate and carboxymethyl cellulose film (Seprafilm®, Genzyme, Baxter International Inc, Unterschleißheim, Germany) and the starch potato-based powder (4DryField®). It can be reasonably deduced that the correct application of the product is an important factor in the avoidance of this adverse effect.

In relation to postsurgical pain, the surgical trauma itself, the use of pneumoperitoneum with CO_2,_ and the peritoneal administration of substances, result in the initiation of an inflammatory reaction that may result in the experience of pain [37,38]. This is a physiological response that occurs simultaneously during the time the antiadhesion agent interacts with the surrounding tissue to achieve its cytoprotective effect on mesothelial surfaces. It is estimated that the laparoscopy itself is responsible for most of the upper and lower abdominal discomfort and the sensation of pressure or pain at the tip of the shoulder [38]. Few studies report on the impact on postoperative pain that may be added by the agent applied during surgery. In the present analysis, following laparoscopic surgery and the administration of the novel HA-based gel, up to 63.33% of study participants reported experiencing these types of discomforts. This proportion is similar to that observed in another study by our research group using potato starch-based powder [39], but lower than the (up to) 80% reported after gynecological endoscopic surgery without the addition of an anti-adhesive agent [39]. This suggests that, in both studies, the postoperative pain was related to the procedure itself and not to the application of the product.

On the other hand, the following measures have been taken and are suggested to avoid the handling difficulties described in the results: The applicator should be connected to the syringe with a Luer lock to avoid loosening. The rigid applicator can be partially bent and moved to the correct position using strong guiding forceps. The gel can be fixed to the ventral peritoneum by using an axillary intra-abdominal instrument and applying pressure for a few minutes to push the gel onto the peritoneum.

As observed, adverse effects following the use of available antiadhesion agents are rarely documented, or the adverse effects are associated with the procedure rather than the agent [25,26,36]. Conversely, improvements regarding adhesion reduction in a significant proportion (up to 85%) of treated patients are reported [36]. It is also noteworthy that the prevention of postsurgical adhesion formation remains a challenging endeavor [1,9,22,37], and no clinical consensus or guidance is available regarding the most effective anti-adhesion agent [9]. The promissory translational tissue engineering research spectrum will facilitate the elucidation of the mechanisms underlying adhesion formation and other fibrotic reactions. Furthermore, it will contribute to the development of novel molecular inhibitors and pharmacological agents targeting signaling pathways, with the objective of achieving an effective and safe reduction in postsurgical adhesions [21].

## 5. Strengths and Limitations

Similar to other pilot studies, this analysis has some limitations due to its small sample size and single institution and single cohort design, which may limit the generalizability of its results. Nevertheless, in the present study, we have employed an inexpensive inflammatory marker that is typically included in the routine monitoring of surgical procedures. This allows for a straightforward comparison of results across future studies. In addition, the low rate of adverse events observed and low impact on inflammatory parameters, as well as the easy of handling, reflect the feasibility of using this gel after non-bowel DIE surgery.

## 6. Conclusions

The results of the present analysis show a low inflammatory response after the application of the novel self-crosslinked hyaluronic acid gel (HyaRegen^®^). Laboratory parameters showed no unacceptable changes in CRP or leukocyte count parameters. Handling was reported to be satisfactory. In addition, no serious adverse effects occurred, and no complications could be attributed to the product. Further studies on adhesion prophylaxis in DIE surgery should also report on changes in inflammatory markers to avoid misdiagnosis of post-operative infection, which can occur due to misinterpretation of CRP levels.

## Figures and Tables

**Table 1:** Adverse effects after the first laparoscopy for deep-endometriosis surgery and application of anti-adhesion gel.

	Yes N (%)	No N (%)	Absent N (%)	Total N (%)
Pain of any kind and localization	38 (63.33%)	22 (63.33%)	O (0.00%)	60 (100%)
Headache	O (0.00%)	O (0.00%)	O (0.00%)	60 (100%)
Stomach pain	37 (61.67%)	23 (38.33%)	O (0.00%)	60 (100%)
Postoperative pain	38 (63.33%)	22 (36.67%)	O (0.00%)	60 (100%)
Late onset pain	O (0.00%)	O (0.00%)	O (0.00%)	60 (100%)
Signs of Inflammation	1 (1.67%) *	59 (98.33%)	O (0.00%)	60 (100%)
Fever	1 (1.67%) *	59 (98.33%)	O (0.00%)	60 (100%)
Prolonged fever	O (0.00%)	O (0.00%)	O (0.00%)	60 (100%)
Abcess	O (0.00%)	O (0.00%)	O (0.00%)	60 (100%)
Chills	O (0.00%)	O (0.00%)	O (0.00%)	60 (100%)
Redness on the back and mons pubis	O (0.00%)	O (0.00%)	O (0.00%)	60 (100%)
One-side labial swelling	O (0.00%)	O (0.00%)	O (0.00%)	60 (100%)
Delayed healing	O (0.00%)	O (0.00%)	O (0.00%)	60 (100%)
Urinary tract infection	1 (1.67%) *	59 (98.33%)	O (0.00%)	60 (100%)
Infection of any kind of localization	1 (1.67%) *	59 (98.33%)	O (0.00%)	60 (100%)
Peritonitis	O (0.00%)	O (0.00%)	O (0.00%)	60 (100%)
Anaphylaxis	O (0.00%)	O (0.00%)	O (0.00%)	60 (100%)
Ichting	O (0.00%)	O (0.00%)	O (0.00%)	60 (100%)
Rash	O (0.00%)	O (0.00%)	O (0.00%)	60 (100%)
Redness	O (0.00%)	O (0.00%)	O (0.00%)	60 (100%)
Gastrointestinal symptoms	O (0.00%)	O (0.00%)	O (0.00%)	60 (100%)
Vomiting	O (0.00%)	O (0.00%)	O (0.00%)	60 (100%)
Ileus	O (0.00%)	O (0.00%)	O (0.00%)	60 (100%)
Increased CRP-parameter	23 (33.38%)	38 (63.33%)	2 (3.33%)	60 (100%)
Increased leucocytes-parameters	O (0.00%)	O (0.00%)	O (0.00%)	60 (100%)
Increased platelets-parameters	O (0.00%)	O (0.00%)	O (0.00%)	60 (100%)
Fluctuating glucose levels	O (0.00%)	O (0.00%)	O (0.00%)	60 (100%)

* The same patient.

**Table 2:** CRP levels before and after the first laparoscopy.

CRP (mg/dL)	Mean ± SD	≤0.5 N (%)	>0.5–≤1.0 N (%)	1.0–10.0 N (%)	≥10.0 N (%)	Absent N (%)	Total N (%)
Before FLL	0.98 ± 1.46	53 (88.33%)	2 (3.33%)	4 (6.67%)	O (0.00%)	1 (1.67%)	60 (100%)
After FLL	1.03 ± 1.29	33 (55.00%)	11 (18.33%)	14 (23.33%)	O (0.00%)	2 (3.33%)	60 (100%)
*p* value	<0.001						
Values in mg/dL. FLL: first look laparoscopy							

**Table 3:** Leukocyte levels before and after the first laparoscopy.

Leucocytes (Tsd/μL)	Mean ± SD	≤4.0 N (%)	4.0–11.0 N (%)	>11.0 N (%)	Absent N (%)	Total N (%)
Before FLL	6.03 ± 1.91	7 (11.67%)	51 (85.00%)	1 (1.67%)	1 (1.67%)	60 (100%)
After FLL	9.15 ± 2.61	O (0.00%)	44 (73.33%)	14 (23.33%)	2 (3.33%)	60 (100%)
*p* value	<0.001					
Values in Thous/μL. FLL: first look laparoscopy						

## Data Availability

Data is unavailable due to privacy or ethical restrictions.
